# Mediterranean diet for cardiovascular disease: an evidence mapping study

**DOI:** 10.1017/S1368980024000776

**Published:** 2024-04-11

**Authors:** Zi-ling Cai, Liao-yao Wang, Bing-yue Zhang, Ai-song Zhu

**Affiliations:** 1 Basic Medical College, Zhejiang Chinese Medical University, Hangzhou 310053, People’s Republic of China; 2 Section of Integrative Medicine, Zhejiang Provincial People’s Hospital, Hangzhou 310053, People’s Republic of China; 3 Key Laboratory of Blood-stasis-toxin syndrome of Zhejiang Province, Hangzhou 310053, People’s Republic of China; 4 Zhejiang Engineering Research Center for ‘Preventive Treatment’ Smart Health of Traditional Chinese Medicine, Hangzhou 310053, People’s Republic of China

**Keywords:** Mediterranean diet, CVD, A Measurement Tool to Assess Systematic Reviews 2, Grading of Recommendations Assessment, Development and Evaluation, Systematic review

## Abstract

**Objective:**

This study aimed to evaluate the methodological quality of existing meta-analyses (MA) and the quality of evidence for outcome indicators to provide an updated overview of the evidence concerning the therapeutic efficacy of the Mediterranean diet (MD) for various types of CVD.

**Design:**

We conducted comprehensive searches of PubMed, Cochrane Library, and Embase databases. The quality of the MA was assessed using the A Measurement Tool to Assess Systematic Reviews 2 (AMSTAR 2) checklist, while the Grading of Recommendations Assessment, Development and Evaluation (GRADE) evidence evaluation system was employed to evaluate the quality of evidence for significant outcomes.

**Setting:**

The CVD remains a significant contributor to global mortality. Multiple MA have consistently demonstrated the efficacy of medical interventions in managing CVD. However, due to variations in the scope, quality and outcomes of these reviews, definitive conclusions are yet to be established.

**Participants:**

This study included five randomized trials and twelve non-randomized studies, with a combined participant population of 716 318.

**Results:**

The AMSTAR 2 checklist revealed that 54·55 % of the studies demonstrated high quality, while 9·09 % exhibited low quality, and 36·36 % were deemed critically low quality. Additionally, there was moderate evidence supporting a positive correlation between MD and CHD/acute myocardial infarction, stroke, heart failure, cardiovascular events, coronary events and major adverse cardiovascular events.

**Conclusions:**

This study indicates that although recognizing the potential efficacy of MD in managing CVD, the quality of the methodology and the evidence for the outcome indicators remain unsatisfactory.

## Graphical abstract

**Figure:**
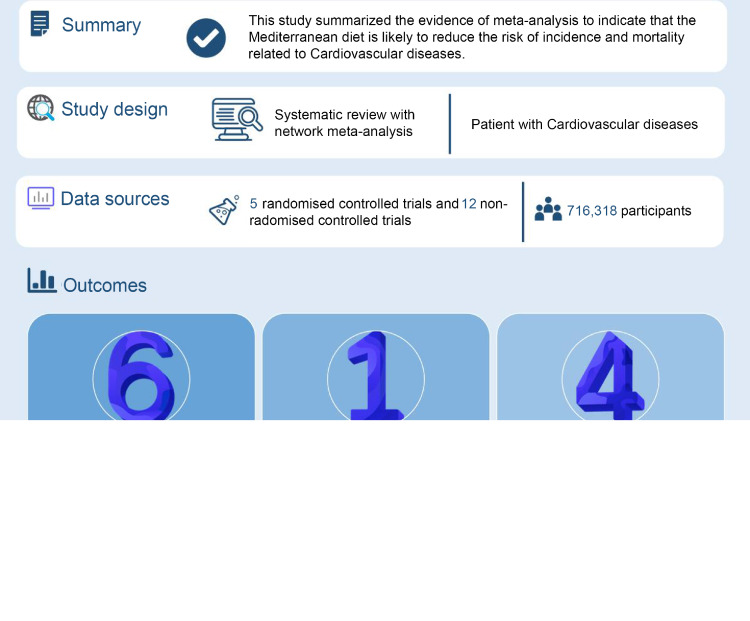

The prevalence of CVD continues to rise globally, making it a leading cause of death and attracting significant global attention^(1–3)^. According to the Global Burden of Disease study^(3)^, CVD imposes a substantial economic burden on the world. Therefore, it is imperative to explore effective strategies for preventing and managing CVD, reducing its incidence and improving prognosis. In recent years, there has been a growing focus on the potential CVD benefits of the Mediterranean diet (MD). The MD is widely acknowledged as a healthy dietary pattern in countries such as Portugal, Spain and Greece within the Mediterranean region. It emphasizes consuming high amounts of monounsaturated fats, primarily derived from virgin and extra virgin olive oil, along with fruits, vegetables, nuts/legumes and grains as primary sources of fats. Additionally, it promotes moderate intake of dairy products, fish, poultry and alcohol while limiting red and processed meat consumption^(4)^. This dietary pattern is characterized by low saturated fat intake while providing essential vitamins and minerals through increased consumption of fruits, vegetables and olive oil^(5)^.

Recent research suggests that adhering to the MD significantly reduces the risk of CVD compared with other structured diets^(6)^. For instance, the low-fat diet focuses on reducing overall fat intake to achieve lower daily energy consumption. However, when compared with the MD approach which includes essential fatty acids in its composition, this low-fat diet may lack certain nutrients leading to an increased incidence of chronic diseases like hyperlipidaemia and CHD^(7,8)^. Additionally, two randomized controlled trials^(7,9)^ and several observational studies^(10–13)^ have demonstrated that a higher adherence to MD is associated with reduced mortality and morbidity from CVD. However, several meta-analyses (MA) of randomized control trials (RCT) have indicated uncertainty regarding the potential beneficial effects of the MD on cardiovascular mortality. Inconsistent conclusions may be attributed to unrecorded variations in saturated fat intake as well as other factors such as unsaturated fat content or methodological biases^(14–16)^.

MA have been conducted to evaluate the efficacy of MD in preventing CVD; however, inconsistent conclusions have been drawn due to variations in scope, quality and outcomes. Some studies^(17)^ suggest that the MD significantly prevents CVD deterioration, while others^(18)^ argue that its role in risk reduction remains uncertain. To overcome limitations of individual systematic reviews, a comprehensive overview of existing evidence is necessary. Therefore, this study aims to comprehensively summarize the available evidence on the effectiveness of the MD for CVD.

## Methods

This review was registered on PROSPERO (CRD42023416139) and followed the guidance from the Preferred Reporting Items for Overviews of Reviews (PRIOR) statement^(19)^.

### Data sources and searches

We searched PubMed, Embase and the Cochrane Library from inception to 3 March 2023. The search strategy was as follows: (‘Diet, Mediterranean’ [Mesh] OR (Diet, Mediterranean [Title/Abstract]) OR Mediterranean*) AND (‘Meta-Analysis’ [Publication Type] OR ‘Meta-Analysis as Topic’ [Mesh] OR (Meta analys* [Title/Abstract]) OR Systematic review* [Title/Abstract]). In addition, the reference lists of related literatures were also examined to ensure the comprehensiveness of the search. Online supplementary material, Supplemental Table S1, provides details of the search strategy.

### Inclusion criteria

The inclusion criteria are as follows:

1. Population: participants diagnosed with CVD
2. Intervention: MD, including, but not limited to, fruits, vegetables, legumes, cereals, meat, dairy products, fish, alcohol and healthy fats
3. Control: a diet corresponding to the intervention applied as a comparison, such as a low-fat diet, dietary advice according to National Cholesterol Education Program (NCEP) guidelines or prudent western diet advice provided by attending physicians
4. Outcome: report at least one of the following outcomes – CVD incidence, mortality, major cardiovascular events (MACE) or other CVD-related outcomes
5. Study design: MA of RCT, case–control and cohort studies

### Exclusion criteria

The exclusion criteria included the following:

1. Protocols, meeting abstracts and MA without full text
2. Literature written in a language other than English
3. Diet patterns did that were not explicitly identified as the MD

### Study selection

The retrieved literature was imported into Endnote X9 software, and duplicate results were eliminated. Following the selection criteria, two independent researchers (Z.C. and L.W.) screened titles and abstracts to select relevant studies. The full text of all articles was obtained for a detailed evaluation based on the inclusion/exclusion criteria. Finally, we shortlisted the relevant studies that we obtained. Any disagreements were resolved through discussion between the two researchers or by reaching a consensus within the group.

### Data extraction

A standardized form was utilized for data extraction from all included MA, encompassing the subsequent details: primary author, publication year, research type, number of incorporated literature sources, participants’ characteristics, treatment and control interventions or exposures, outcome measures, quality assessment tools employed, primary or secondary prevention focus areas, and key findings obtained. Data were independently extracted by two researchers (Z.C. and L.W.), with any discrepancies resolved through consensus after thorough re-evaluation by all authors.

### Assessment of the methodological quality

We utilized the A Measurement Tool to Assess Systematic Reviews 2 (AMSTAR 2)^(20)^ to evaluate the methodological quality of the included MA. AMSTAR 2 comprises a total of sixteen items, with seven critical domains (items 2, 4, 7, 9, 11, 13 and 15) that significantly impact the review’s validity and conclusions. The overall confidence in the review’s findings was categorized as ‘high’, ‘moderate’, ‘low’ or ‘critically low’. The assessment process involved independent evaluation by two authors with any discrepancies resolved through consensus among all authors.

### Assessment of the evidence quality

The Grading of Recommendation, Assessment, Development and Evaluation (GRADE) system^(21)^ was utilized by two independent authors (L.W. and Z.C) to evaluate the quality of evidence for each outcome across four levels: high, moderate, low and very low quality. We assessed the evidence based on five key aspects: risk of bias, inconsistency, indirectness, imprecision and publication bias. Any discrepancies between the two authors were resolved through a final consensus among all contributors. Reviewer disagreements were addressed through thorough discussion.

## Results

### Study selection

**Fig. 1:**
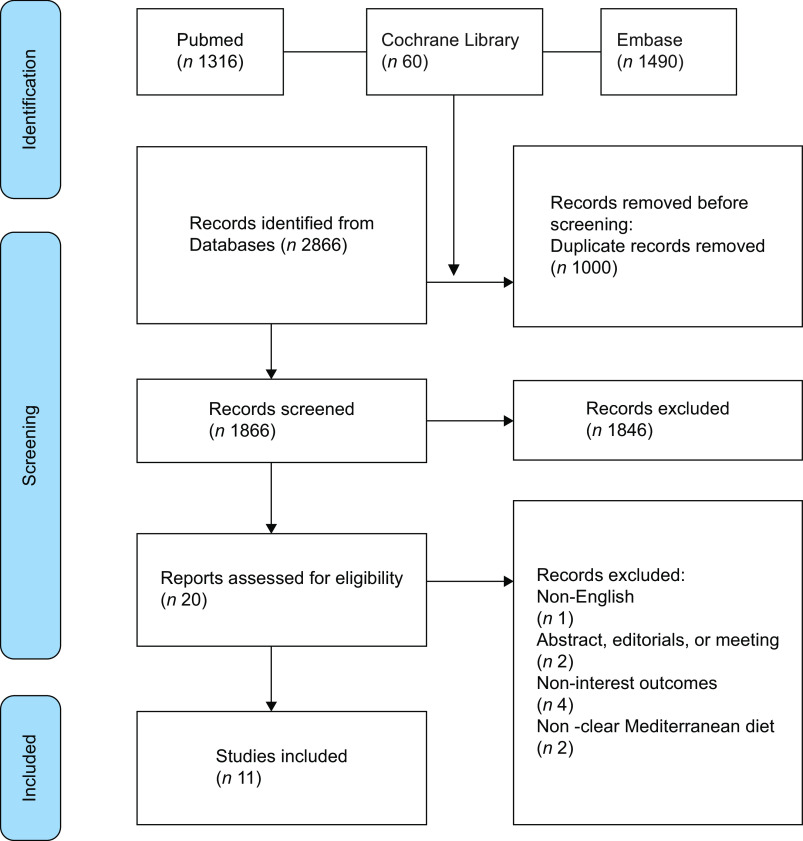
Preferred Reporting Items for Systematic Reviews and Meta-Analyses flow diagram. The figure depicts the screening process of the studies.

We identified a total of 2866 published studies and subsequently screened their titles to remove citations. After this process, we selected twenty potentially eligible studies for closer scrutiny by retrieving the full text. Ultimately, our analysis included eleven MA^(17,22–31)^. The study selection process is shown in Fig. 1.

### Characteristics of the included studies

**Fig. 2:**
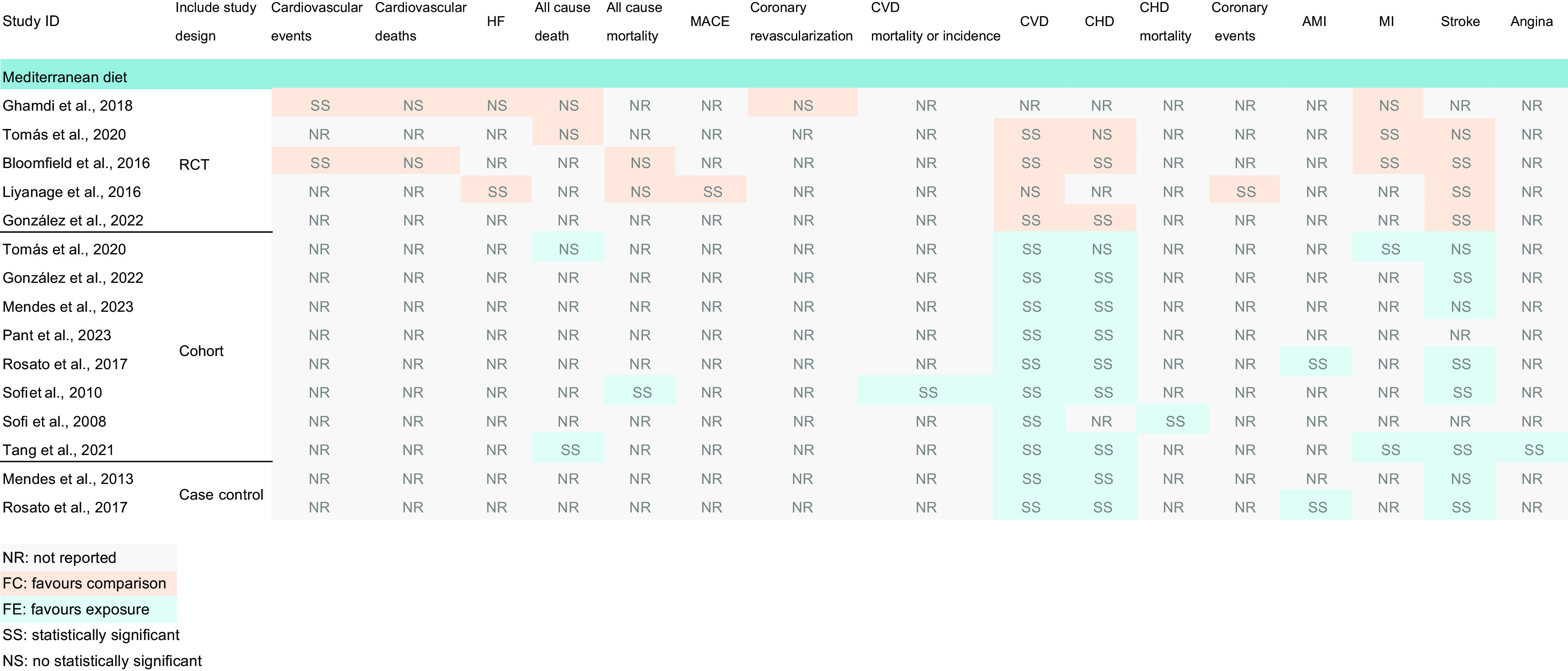
Evaluation outcomes of the included meta-analyses. Abbreviations: HF, heart failure; AMI, acute myocardial infarction; MI, myocardial infarction; MACE, major adverse cardiovascular events.

We conducted a comprehensive search of 2866 relevant articles and, after removing duplicates and reviewing the title and abstract, selected a final set of twenty articles. Ultimately, we included eleven articles that encompassed a total of 7987–1 175 416 patients with CVD. Based on the original outcomes of the included literature, the MA included in this study were published between 2008 and 2023 and focused on the associations between MD and various cardiovascular outcomes: cardiovascular events (*n* 1)^(22)^, CVD (*n* 7)^(23,26–31)^, CHD (*n* 3)^(23,27,28)^, stroke (*n* 4)^(23,25,26,29)^, myocardial infarction (MI) (*n* 1)^(23)^, CHD/acute myocardial infarction (*n* 1)^(29)^, heart failure (*n* 1)^(25)^ and MACE (*n* 2)^(24,25)^. The primary studies originated from Spain, Australia and Italy, and most studies included both male and female participants. Detailed information about the included MA can be found in Table 1, Fig. 2 and online supplementary material, Supplemental Figs. S1–S2.

**Table 1:** Characteristics of the included meta-analyses

Study	Databases	Age	Include study design	Exposure *v*. outcomes	Treatment *v*. control	Primary or secondary prevention	Effect value type
Ghamdi *et al*.^(22)^	6	41–67	RCT	/	MD *v*. low-fat diet/NCEP guideline dietary	Primary	RR
Tomás *et al*.^(23)^	3	16–92	Prospective cohort studies and RCT	MD *v*. ①②③④⑤	MD/Indo-MD/MD + EVOO + nuts *v*. step I diet of the AHA/low-fat diet	Secondary	RR/OR/HR
Bloomfield *et al*.^(24)^	3	19–80	Case control, prospective cohort studies and RCT	MD + fat intake *v*. ②③④⑤	MD + EVOO/nuts *v*. low-fat diet	Primary and secondary	HR/RR
Liyanage *et al*.^(25)^	3	41–67	RCT	**/**	High bread, vegetables, fish and less meat/MD/ MD + extra virgin olive oil/MD + nuts *v*. prudent western diet/NCEP guidelines dietary/sensible eating/low-fat diet	Primary and secondary	RR
Martínez-González *et al*.^(26)^	2	18–96	Prospective cohort studies and RCT	Olive oil consumption *v*. ②③⑤	MD + EVOO/ MD + nuts *v*. low-fat diet	Primary	RR/OR/HR
Mendes *et al*.^(27)^	3	19–83	Prospective cohort studies and case–control studies	Legume *v*. ②③⑤	**/**	**/**	RR/OR/HR
Pant *et al*.^(28)^	5	≥18	Prospective cohort studies	MD *v*. ②③④⑤⑥⑦	**/**	Primary	RR/OR/HR
Rosato *et al*.^(29)^	1	1–92	Case–control or cohort studies	MD *v*. ②③⑤⑧	**/**	Primary	RR/OR/HR
Sofi *et al*.^(30)^	6	29–69	Prospective cohort studies	MD *v*. ②③⑤	**/**	Primary	RR
Sofi *et al*.^(31)^	4	20–90	Prospective cohort studies	MD *v*. ⑨⑩	**/**	Primary	RR
Tang *et al*.^(17)^	6	20–86	Prospective cohort studies	MD *v*. ②③④⑤⑨	**/**	**/**	HR

RCT, randomized controlled trial; MD, Mediterranean diet; NCEP, national cholesterol education program; EVOO, extra virgin olive oil; AHA, American Heart Association; RR, Relative risk; HR, Hazard ratio; ①, all-cause death; ②, cerebrovascular disease; ③, CHD; ④, myocardial infarction; ⑤, stroke; ⑥, heart failure; ⑦, major adverse cardiovascular events; ⑧, acute myocardial infarction; ⑨, CVD mortality; ⑩, CHD mortality.

### Assessment of the methodological quality

**Fig. 3:**
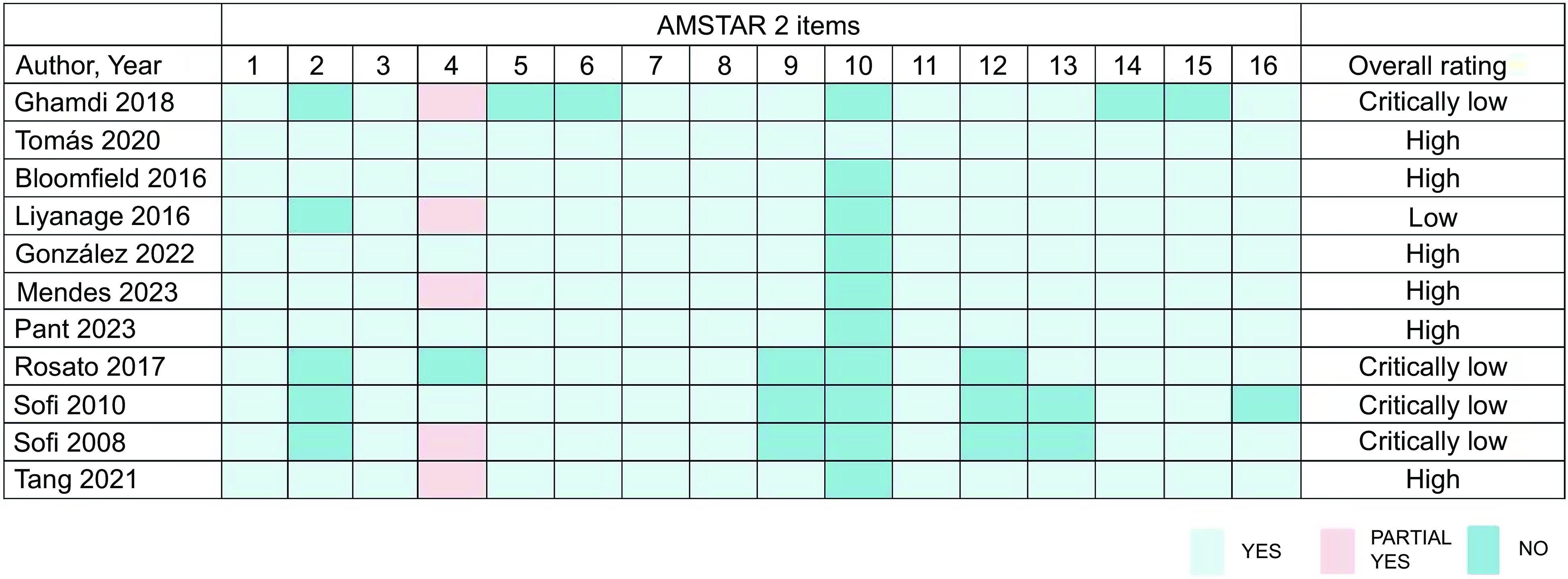
Evaluation results of the included meta-analyses by A Measurement Tool to Assess Systematic Reviews 2.

According to the AMSTAR 2 checklist, we assessed the methodological quality of eleven MA, and six of MA^(18,24,26–29)^ were rated as high, while the others were rated as low or extremely low. All included MA specified their inclusion and exclusion criteria, including population, interventions, comparators and outcomes, and used appropriate MA methods to analyse the results. In low-quality or critically low-quality MA, critical flaws included a lack of protocol registered before the commencement of the review (*n* 5), a lack of comprehensive literature search (*n* 1), insufficient technique for assessing and explaining the risk of bias (*n* 3) and lack of assessment of the presence and likely impact of publication bias (*n* 1). The rating of the overall confidence in the results of the evaluation by AMSTAR 2 is shown in Fig. 3.

### Evidence quality

**Fig. 4:**
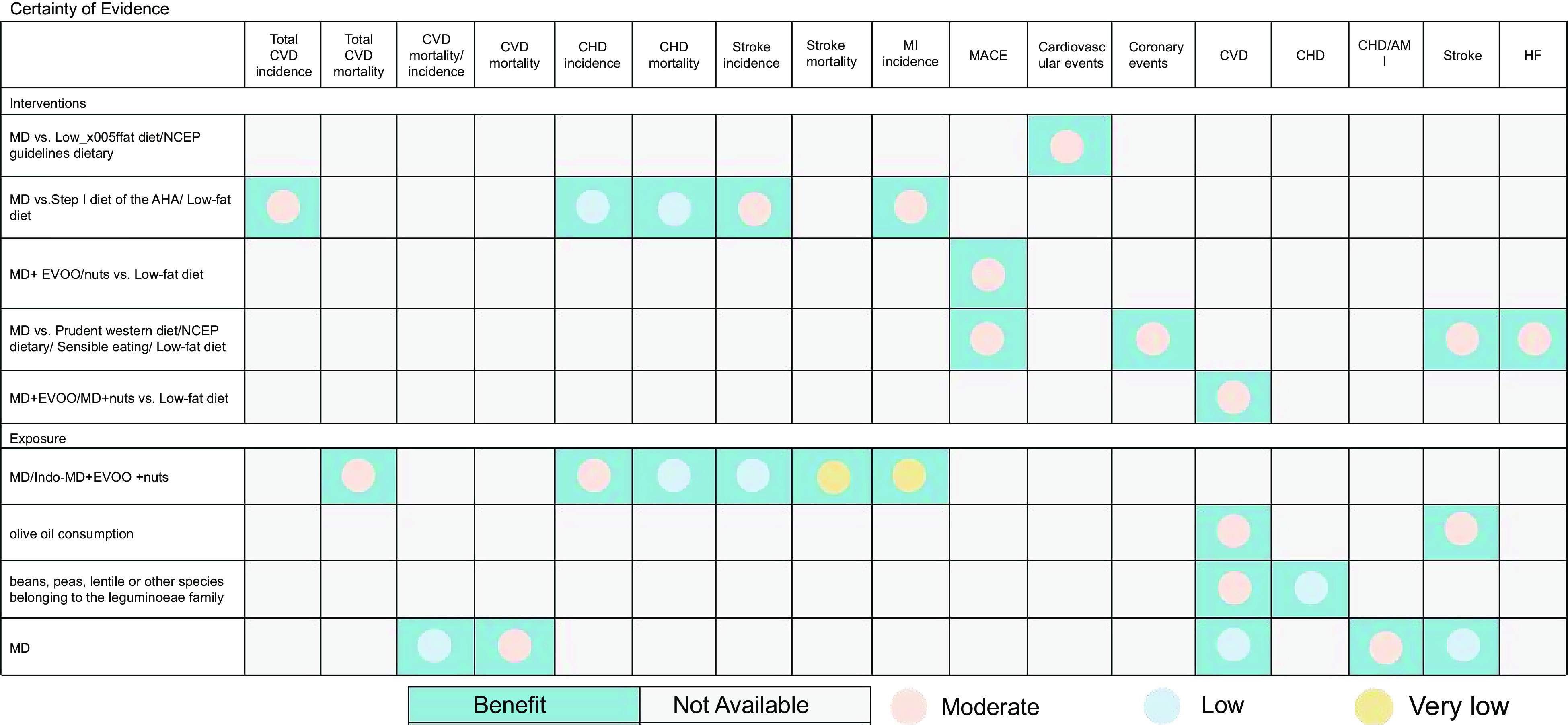
Evidence mapping of availability and appraisal of certainty of the evidence. Abbreviations: HF, heart failure; AMI, acute myocardial infarction; MI, myocardial infarction; MACE, major adverse cardiovascular events.

This overview focused on three outcomes: the effect of the MD on CVD incidence compared with other diets, the effect of the MD on CVD mortality compared with other diets and the effect of the MD on the recurrence rates of CVD compared with other diets. The evidence synthesis for each outcome according to the GRADE system is summarized below and in Fig. 4. There were twenty (60·60 %) moderate-quality evidences, eleven (33·33 %) pieces of low-quality evidences and two (6·06 %) very low-quality evidences.

### Effect of the MD on the incidence of CVD

#### Total CVD incidence

Six MA^(23,26–29,31)^ reported total CVD morbidity. The MA published by Becerra-Tomás *et al*.^(23)^ included three RCT studies that showed that MD was associated with lower total CVD morbidity (Relative risk (RR) = 0·62, 95 % CI: 0·50, 0·78; moderate quality). The MA published by Martínez-González *et al*.^(26)^ included one RCT study (RR = 0·73, 95 % CI: 0·58, 0·92; moderate quality) and nine prospective cohort studies (RR = 0·83, 95 % CI: 0·74, 0·94; moderate quality) showing that MD was associated with a lower incidence of CVD when compared with a low-fat diet. The MA published by Mendes *et al*.^(27)^ included five case–control studies showing that MD was associated with a reduced risk of CVD (RR = 0·72, 95 % CI: 0·60, 0·87; low quality). Pant *et al*.^(28)^ published a MA that included four prospective cohort studies showing that MD was associated with a reduced incidence of CVD (Hazard ratio (HR) (F) = 0·76, 95 % CI: 0·72, 0·81; low quality; HR (M) = 0·78, 95 % CI: 0·72, 0·83; low quality). The MA published by Rosato *et al*.^(29)^ included five case–control studies, which demonstrated a protective effect of MD on the risk of CVD development (RR = 0·81, 95 % CI: 0·74, 0·88; low quality). Another MA published by Sofi *et al*.^(31)^ included three observational studies that demonstrated a protective effect of MD on total CVD incidence (RR = 0·90, 95 % CI: 0·87, 0·93; low quality).

#### CHD incidence

Three MA^(23,27,28)^ reported on the incidence of CHD. The MA published by Becerra-Tomás *et al*.^(23)^ included one RCT study (RR = 0·48, 95 % CI: 0·33, 0·71; low quality) and seven observational studies (RR = 0·73, 95 % CI: 0·62, 0·86; moderate quality) reporting that MD was associated with a reduced incidence of CHD. The MA published by Mendes *et al*.^(27)^ included twenty-one prospective cohort studies (RR = 0·92, 95 % CI: 0·87, 0·98; low quality) and five case–control studies (RR = 0·72, 95 % CI: 0·60, 0·87; low quality), which demonstrated that MD reduced the incidence of CHD. Another MA published by Pant *et al*.^(28)^ included four prospective cohort studies (HR = 0·75, 95 % CI: 0·65, 0·87; low quality) showing that MD was associated with a reduced incidence of CHD.

#### Stroke incidence

Four MA^(23,25,26,29)^ reported on stroke incidence. The MA published by Becerra-Tomás *et al*.^(23)^ included one RCT study (RR = 0·58, 95 % CI: 0·42, 0·81; moderate quality) and five observational studies (RR = 0·80, 95 % CI: 0·71, 0·90; moderate quality), which demonstrated that MD was superior to the American Heart Association/low-fat diet of the step I diet in reducing the incidence of stroke. Liyanage *et al*.^(25)^ showed that MD reduced the incidence of stroke compared with a cautious western diet/NCEP guideline diet/rational diet/low-fat diet (RR = 0·65, 95 % CI: 0·48, 0·88; moderate quality). In addition, Martínez-González *et al*.^(26)^ showed that consumption of MD and olive oil had a reducing effect on the risk of stroke (RR = 0·74, 95 % CI: 0·61, 0·91; moderate quality). Rosato *et al*.^(29)^ published the MA including twenty-four cohort studies (RR = 0·77, 95 % CI: 0·67, 0·90; moderate quality) and five case–control studies (RR = 0·12, 95 % CI: 0·03, 0·46; moderate quality) and demonstrated a protective effect of MD on the risk of stroke.

#### MI incidence

One MA^(23)^ reported the incidence of myocardial infarction. The MA published by Becerra-Tomás *et al*.^(23)^ included two RCT studies (RR = 0·65, 95 % CI: 0·49, 0·88; moderate quality) and two observational studies (RR = 0·73, 95 % CI: 0·61, 0·88; very low quality) demonstrating that MD reduced the incidence of MI compared with the American Heart Association step I diet or low-fat diet.

#### CHD/acute myocardial infarction incidence

One MA^(29)^ reported CHD/acute myocardial infarction incidence. The MA published by Rosato *et al*.^(29)^ included twenty-four cohort studies (RR = 0·74, 95 % CI: 0·66, 0·83; moderate quality) and five case–control studies (RR = 0·41, 95 % CI: 0·18, 0·98; moderate quality), which showed that MD reduced the incidence of CHD/acute myocardial infarction.

#### HF incidence

One MA^(25)^ reported on heart failure incidence. The MA published by Liyanage *et al*.^(25)^ included two RCT studies that demonstrated that MD reduced the incidence of heart failure (RR = 0·30, 95 % CI: 0·17, 0·56; moderate quality).

### Effect of the MD on mortality of CVD

#### Total CVD mortality

Three MA^(23,30,31)^ reported total CVD mortality. The MA published by Becerra-Tomás *et al*.^(23)^ included twenty-one observational studies (RR = 0·79, 95 % CI: 0·77, 0·82; moderate quality) suggested that MD reduced total CVD mortality. Sofi *et al*.^(30,31)^ published two MA supporting the conclusion that MD reduced total mortality from CVD (RR = 0·90, 95 % CI: 0·87, 0·93; low quality (2008); RR = 0·91, 95 % CI: 0·87, 0·95; moderate quality (2010)).

#### CHD mortality

One MA^(23)^ reported on CHD mortality. The MA published by Becerra-Tomás *et al*.^(23)^ included one RCT study (RR = 0·33, 95 % CI: 0·13, 0·86; low quality) and six observational studies (RR = 0·73, 95 % CI: 0·59, 0·89; low quality), which demonstrated that MD was superior to American Heart Association/low-fat diet class I in lowering CHD mortality.

#### Stroke mortality

One MA^(23)^ reported stroke mortality. The MA published by Becerra-Tomás *et al*.^(23)^ included four observational studies showing that MD improved stroke mortality (RR = 0·87, 95 % CI: 0·80, 0·96; very low quality).

### Effect of the MD on the recurrence rates of CVD

#### Recurrence rates of cardiovascular events

One MA^(22)^ reported recurrence rates of cardiovascular events. A medical review published by Al-Ghamdi *et al*.^(22)^ included two RCT studies that demonstrated a superior effect of the MD on recurrence rates of cardiovascular events compared with a low-fat diet/NCEP guideline diet (RR = 0·83, 95 % CI: 0·72, 0·97; moderate quality).

#### Recurrence rates of MAC

Two MA^(24,25)^ reported recurrence rates of MACE. The MA published by Bloomfield *et al*.^(24)^ included one RCT study showing that MD significantly improved recurrence rates of MACE (HR = 0·71, 95 % CI: 0·56, 0·90; moderate quality). Another MA published by Liyanage *et al*.^(25)^ included three RCT studies, also showing that MD significantly improved the recurrence rates of MACE compared with prudent western diet/NCEP guidelines dietary/sensible eating/low-fat diet (RR = 0·69, 95 % CI: 0·55, 0·86; moderate quality).

#### Recurrence rates of coronary events

One MA^(25)^ reported the recurrence rates of coronary events. The MA published by Liyanage *et al*.^(25)^ included three RCT studies, which showed that MD reduced the recurrence rates of coronary events (RR = 0·65, 95 % CI: 0·50, 0·85; moderate quality).

## Discussion

### Overall findings

This review presents a comprehensive overview of the current body of systematic reviews investigating the association between MD and CVD, encompassing incidence, mortality and recurrence rates. A total of eleven systematic reviews were identified, including MA that examined the relationship between MD and CVD incidence, mortality or cardiovascular outcomes.

These systematic reviews were published between 2008 and 2023, addressing various research questions related to the topic at hand. The methodological quality and evidence quality of eleven MA on MD for CVD were assessed using AMSTAR 2 and the GRADE system, respectively. The assessment conducted by AMSTAR 2 revealed that the evidence quality was categorized as high in 54·55 % of cases, moderate in 9·09 % of cases and critically low in 36·36 % of cases. Additionally, six studies^(15,21,22,24–26)^ provided a protocol and had been registered prior to their execution. Failure to register may lead to significant deviations from the expected research process.

Only one study^(23)^ reported the sources of funding, and the absence of this standard may compromise the credibility of research results due to potential conflicts of interest. Therefore, it is essential to register MA of MD for the treatment of CVD and report their treatment regimens prior to study commencement. The GRADE system resulted in a downgrade of the evidence quality for all included outcomes. This comprehensive overview, consisting of eleven studies and encompassing thirty-three outcomes, revealed that the overall quality of evidence was deemed moderate (60·60 %), with low and very low levels accounting for 39·4 %, while no high-quality evidence was identified. Notably, significant evidence predominantly focused on CVD, CHD and stroke. Despite recognizing the potential efficacy of MD in managing CVD, it is important to acknowledge that the strength of evidence across all outcomes remains unsatisfactory.

### Comparison with previous studies

Although our overview represents the first comparative assessment of the effectiveness of MD in treating CVD, a recent Cochrane review conducted by Rees *et al*.^(18)^ further investigated the impact of the MD on MACE, both in terms of randomized trials and cardiac metabolism outcomes. While aligning with our own findings, the Cochrane review did not observe any effects of the MD on CVD outcomes in non-randomized observational studies. In contrast, our study incorporates various research designs and evaluates their efficacy in managing CVD. Additionally, supporting healthy lifestyle behaviours, the 2020 U.S. Preventive Services Task Force (USPSTF) guideline^(32)^ provides recommendations for interventions targeting individuals with cardiovascular risk factors, emphasizing a nutritious diet and regular exercise. Our overview strengthens these recommendations by providing robust evidence that supports the preventive role of the MD against CVD within this population.

### Suggestions for future research

The primary objective of CVD treatment is to achieve clinically significant reduction in symptoms experienced by patients. Current research has demonstrated that adherence to a healthy diet, as supported by studies conducted by Martinez-Gonzalez *et al*.^(33)^, Du *et al*.^(34)^ and Qin *et al*.^(35)^, can effectively prevent numerous cases of CVD within general populations. Recognizing the crucial role of diet in determining disease risk, the WHO recommends dietary modifications that involve maintaining energy balance, limiting the intake of saturated and trans fats while increasing consumption of unsaturated fats, incorporating more fruits and vegetables into one’s diet, and reducing sugar and salt intake – such as following the MD^(36)^. The efficacy of MD in managing CVD among patients has been confirmed through studies like Rees *et al*.’s investigation^(18)^. Arguably, MD stands out as the most extensively researched and evidence-based dietary approach not only for preventing CVD but also other chronic diseases. It serves as a benchmark for healthy eating habits with particular value.

The primary advantage attributed to MD lies in its synergistic effects on various cardioprotective nutrients and foods, as observed by Jacobs *et al*.^(37)^. Consequently, MD presents itself as a potentially significant adjunctive treatment option for individuals with CVD. However, future research should focus on carefully standardizing MD protocols to explore its effects across different stages of CVD. Preliminary data suggest potential benefits related to incidence rates and mortality associated with CVD along with cardiovascular events. Furthermore, methodologically rigorous studies with sufficient sample sizes are necessary for each MD program while consistently reporting on defined core outcomes among patients diagnosed with CVD.

### Strengths and limitations

A comprehensive assessment of the effectiveness of MD in patients with CVD is currently lacking. Therefore, we have synthesized and compared the efficacy of MD programs evaluated in eleven MA. To our knowledge, this overview of MA represents the first comprehensive summary of available evidence on MD for CVD. We assessed the quality of reviews against all sixteen domains of AMSTAR 2 checklist, which offers a wider range of applications and more rigorous evaluation methods than its predecessor^(20,38)^. To determine the strength of evidence, we evaluated significant outcomes using the GRADE system. Most evidence from included studies was considered moderate quality. Thus, our results confirmed moderate-certainty evidence for the efficacy of MD program. These findings are crucial to patients who may be sceptical about potential benefits from MD to improve CVD outcomes.

However, there are certain limitations in this study. Firstly, included studies were limited in number, and some effects on CVD outcomes such as hypertension^(39)^, inflammatory markers with CVD^(40)^ and cardiovascular risk factors^(41)^ did not meet inclusion criteria. Although not evaluated in this study, potential effects cannot be fully ruled out. Furthermore, the inclusion of RCT, non-RCT and observational studies may increase bias risks. Lastly, the results suggested possible benefits for CVD among those on a MD; however, evidence certainty for these outcomes was found to be very low or moderate leading us to conclude that overall evidence is currently insufficient to convincingly support the effects of MD on CVD. Future research should focus on designing more scientific standardized studies.

### Conclusions

Despite the existing gaps in the literature and limited availability of high-quality evidence, the findings of this overview underscore the significance of adopting a MD for individuals with CVD. To establish the true benefits of MD for CVD patients, future research should not only adhere to rigorous methodological requirements but also prioritize enhancing the quality of primary studies and conducting patient-centred MA. The inclusion of higher-quality trials is crucial in providing a more comprehensive understanding of the advantages associated with MD for individuals affected by CVD.

## Supporting information

Cai et al. supplementary material Cai et al. supplementary material

## Supplementary material

For supplementary material accompanying this paper, visit https://doi.org/10.1017/S1368980024000776.
