# Supplementary material for: Mediterranean diet for cardiovascular disease: an evidence mapping study
Source: Public Health Nutr. 2024 Apr 11;27(1):e118. doi: 10.1017/S1368980024000776 (PMC11075113; doi:10.1017/S1368980024000776)
Supplement: Cai et al. supplementary material [file S1368980024000776sup001.pdf]

Supplementary Material

Table S1. Search Strategy

(1) PubMed

Search	Query	Items
#1	"Diet, Mediterranean"[Mesh]	5071
#2	(Diet, Mediterranean[Title/Abstract]) OR Mediterranean*	60305
#3	#1 OR #2	60305
#4	"Meta-Analysis" [Publication Type] OR "Meta-Analysis as Topic"[Mesh]	198744
#5	(Meta analys*[Title/Abstract]) OR Systematic review*[Title/Abstract]	402802
#6	# 4 OR #5	430373
#7	#3 AND #6	1316

(2) Embase

Search	Query	Items
#1	'Diet, Mediterranean'/exp	11592
#2	'Diet, Mediterranean':ab,ti OR Mediterranean*:ab,ti	53881
#3	#1 OR #2	56984
#4	'meta analysis'/exp	272348
#5	'meta analys*':ab,ti OR 'systematic review*':ab,ti	496212
#6	#4 OR #5	541286
#7	#3 AND #6	1489

(3) Cochrane Library

Search	Query	Items
#1	MeSH descriptor: [Diet, Mediterranean] explode all trees	754
#2	(Diet, Mediterranean):ti,ab,kw OR (Mediterranean*):ti,ab,kw	2733
#3	#1 OR #2	2733
#4	MeSH descriptor: [Meta-Analysis as Topic] explode all trees	1439
#5	(Meta analys*):ti,ab,kw OR (Systematic review*):ti,ab,kw	27376
#6	#4 OR #5	27376
#7	#3 AND #6	60

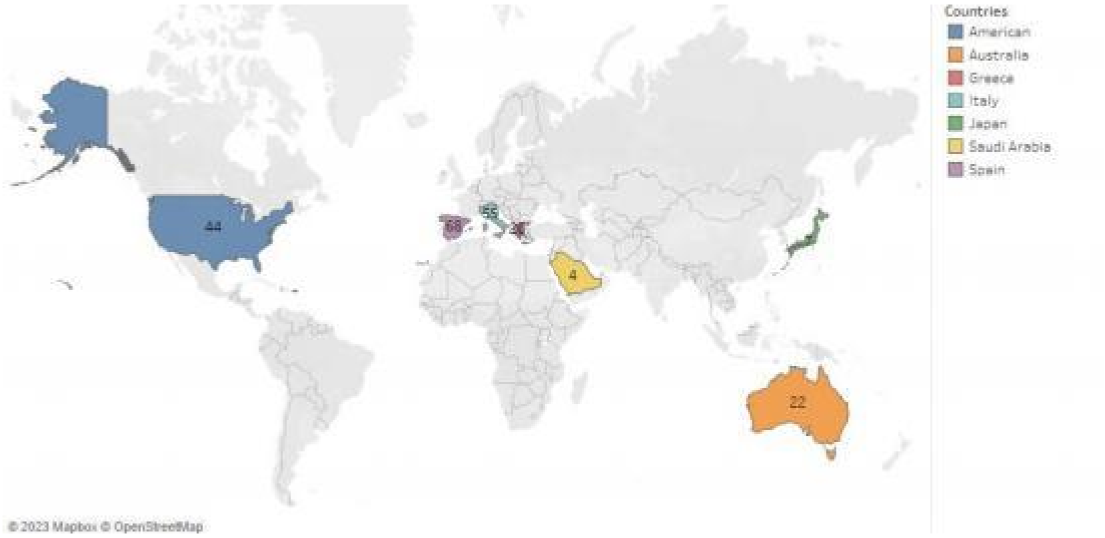

Figure S1. Distribution of publications by country.

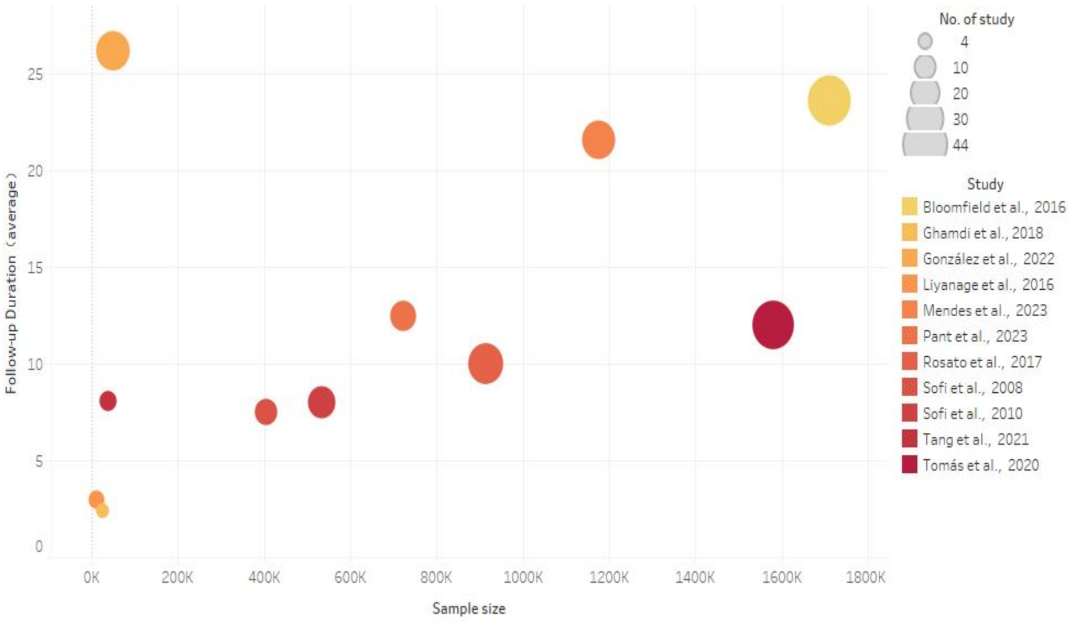

Figure S2. Evidence mapping of MD for cardiovascular disease. Including study, the number of included studies, sample size and follow-up time.
